# Assessment of catchment water resources allocation under climate change in Luwombwa sub-catchment, Zambia

**DOI:** 10.1016/j.heliyon.2024.e39962

**Published:** 2024-10-30

**Authors:** Dickson Mwelwa, Phenny Mwaanga, Alick Nguvulu, Tewodros M. Tena, Gebeyehu Taye

**Affiliations:** a School of Graduate Studies, Copperbelt University, P.O. Box 21692, Kitwe, Zambia; b Department of Environmental Engineering, School of Mines and Mineral Sciences, Copperbelt University, P.O. Box 21692, Kitwe, Zambia; c Department of Geomatics Engineering, School of Mines and Mineral Sciences, Copperbelt University, P.O. Box 21692, Kitwe, Zambia; d Federal Institute for Geosciences and Natural Resources (BGR), Lusaka, Zambia; e Department of Land Resources Management and Environmental Protection, Mekelle University, P.O.Box 231, Mekelle, Ethiopia

**Keywords:** Water resources allocation, Climate change, SSP370 scenario, Water demand

## Abstract

The uncertainty in climate change and high water demand pose pressure on the natural water resources supply. Not only does this require better understanding but also a call for immediate interventions, mitigation and adaptive measures. This study evaluates catchment water resources in the Luwombwa sub-catchment in Zambia through statistical analysis in the downscaling of past, present and future climatic variables from the CMIP6 climatic model. These variables are then integrated into WEAP - a semi-distributed hydrological and water evaluation model - to perform water demand and allocation scenario modelling. Mult-site calibration and validation were conducted on five selected micro-catchments within Luwombwa sub-catchment. The model performance was assessed usng the R^2^, NSE and PBIAS as the objective functions. Satisfactory values of 92 % for R^2^, 82 % for NSE and 6.9 % for PBIAS were achieved. This allowed for scenario modelling on water demand and allocation among competing users. Three future scenarios (2022–2050) were developed from the historical to baseline (1988–2022) and included state of water resources availability under climate change, expansion of irrigation area and impact of dam construction in the sub-catchment. The study reveals a decrease of 20 % in sub-catchment's water availability resulting from 9.3 % (equivalent to 4^o^C) rise in maximum temperature and 4.5 % reduction in rainfall within the entire sub-catchment. This is especially under the persistence of SSP370 climate variability scenario projections downscaled from four GCM models by the year 2050. The study further revealed that the change point for anticipated future climate extremes is likely to occur between 2027 and 2030. The results are indicative of downward trends in streamflow under climate change and socioeconomic development leading to increase in water value and water scarcity. The insights from the study are critical to inform formulation of effective catchment water resources management strategies such as the development of management plans and adapation measures in the face of climate change and the needs for different stakeholders involvement.

## 1 Introduction

The intensity of global changes, notably population growth, urbanisation, expansion in agriculture and climate change, exert a huge pressure on river catchment water resources and the entire hydroecological system [1]. Climate change issues have become a focus of concern for the scientific community and policy makers worldwide. According to Refs. [[2], [3], [4], [5]], projected temperatures and precipitation under different scenarios show that climate change will have different impacts on the various regions of the globe. Africa is poised to be one of the most vulnerable continents to the impacts of climate change due to the diversity of effects and low adaptive capacity [5]. Previous studies, [e.g. [[6], [7], [8]]], indicated that Southern Africa has continued to record rising temperature since 1980s and decrease in rainfall due to interannual variability of the climate driven by El-Nino Southern Oscillation (ENSO) leading to drought conditions.

Recent studies on the Southern African region indicated an increase in temperatures, reduction in streamflow and precipitation under both RCP4.5 and RCP8.5 [[9], [10], [11], [12]], increase in precipitation and evapotranspiration and a decrease in water yield and groundwater recharge under SSP2-4.5 and SSP5-8.5 scenarios of climate change projections [13]. A study by Ref. [14] reported an increase in temperature, a decrease in rainfall and water availability by the end of the century in 2100 at the national level in Zambia. Most of the studies conduced in the region have used an ensemble system from General Circulation Models (GCM) projections in hydrological modeling and they have mainly focused on the main catchments like the Zambezi, Kabompo and Kafue. Very few studies [15,16] have been conducted on smaller but very sensitive catchments like the Luwombwa sub-catchment which houses one of the three active national farm blocks that are critically important to national and regional food security. The studies in Refs. [15,16] used the WEAP model to model the catchment water resources availability to assess the impacts of land use land cover change on hydrological parameters in the rapidly urbanizing Chongwe River catchment. However, the impact of climate change scenarios on small but sensitive catchment water resources availability and allocation among competing users has remained less investigated.

This study aims to fill the gap by assessing the impact of climate change on future catchment water availability in the Luwombwa sub-catchment through the application of the Water resources Evaluation And Planning (WEAP) model coupled with GCM focusing on SSP370 scenario of climate change projection. The overall objective of the study was to estimate the magnitude of climate change for the selected variables – high temperature whose effect is directed to sub-catchment's evaporation losses and rainfall amounts by the year-2050. In the study; effects of past, present and future climatic change under scenario SSP370 statistically downscaled from Coupled Model Intercomparison Project sixth hase (CMIP6) models are simulated on the Luwombwa sub-catchment's water resources including water demand and availability trend analysis [17]. Luwombwa sub-catchment, with average annual rainfall of 1000 mm is among the major rivers systems in the region where the effects of climate change have been felt due to rise in temperature and decrease in rainfall amount [18]. The novelty of this study consists in the detailed analysis of water availability under high resolution for high uncertainty and strong consensus at sub-catchmnet level. The generated new knowledge for water availability under various scenarios could be useful for future water resources planning in respective catchments. The study will contribute towards water resources management strategies and a review of policies at the local and national levels and echoes the United Nations' Sustainable Development Goals (SDGs) 11 and 13: Sustainable cities and communities and Climate action and the African Union's Agenda2063 Goal No. 7: Environmentally sustainable and climate resilient economies and communities.

## 2 Materials and methods

### 2.1 Description of study area

#### 2.1.1 Geography

**Fig. 1:**
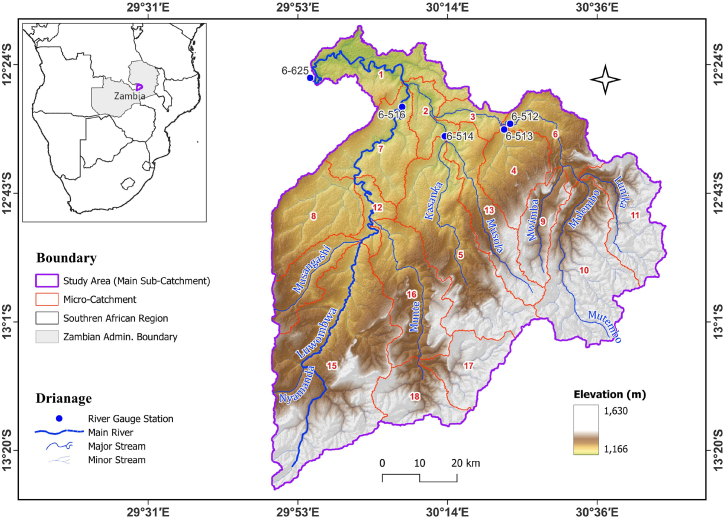
Location of Luwombwa sub-catchment in Zambia and Digital Elevation Model (DEM) of Luwombwa sub-catchment with indication of river drainages, micro-catchments and river gauging stations.

Luwombwa River is a tributary of the Luapula River and has its headwaters located South-West of Serenje town within the Central Province of Zambia in Southern Africa (Fig. 1). The river spans approximately 216 km from its headwaters to the hydrometric station 6–625 just before the confluence with the Luapula River. The Luwombwa River meanders through a catchment area of approximately 7363 km^2^. Its major tributaries include are Mutembo, Munte, Musangashi, and Nyamanda streams. Apart from station 6–625, there are four other hydrometric stations namely 6–512, 6–513, 6–514 and 6–516 in the catchment (Fig. 1).

#### 2.1.2 Climate and Agro-ecological setting

The climate in the catchment is strongly seasonal with average annual precipitation of 1000 mm, average temperature ranges between 13 °C minimum and 27 °C maximum, average relative humidity at 58 % and wind speed at 2 m/s. The catchment is located in Agro-ecological Zone III with seasonality based on the three distinct subtropical climatic seasons experienced in Zambia. The seasons are: a hot, dry season experienced from the month of August to October (ASO) characterised by natural low streamflow, a hot wet season from November to April (NDJFMA) period with peak streamflow and a cool dry season from the month of May to July (MJJ) with average streamflow.

#### 2.1.3 Land use and demographics

Land use is an elaborative depiction of how the population of the region utilises the land and land resources in their socioeconomic activities. The land use land cover types in the study area include forest, grassland, agriculture land, wetlands, and settlements. The respective dominant land use land cover types are forest lands and grasslands covering at least 44 % and 24.7 % of the total catchment area, from which approximately 858 km^2^ cover gazzetted forest reserves in the upper part of the catchment. Agricultural land use includes both rainfed and large-and-small-scale irrigated agriculture. Large scale irrigation depends directly on the Luwombwa River especially during the dry season when there is little or no rainfall. The riparian conditions in the sub-catchment and water resources support wildlife in Kasanka National Park and the envisioned large-scale mechanized farming such as the Nansanga Farm Block. The sub-catchment is poised to form the basis for the socioeconomic development, contributing to national food security and ultimately improving people's livelihood. This will require scientific facts on water resourcse availability to ensure sustainable water allocation among economic sectors and the environment. Large-scale irrigation is one of the most prominent water use in the Luwombwa sub-catchment with at least 80 km^2^ of cropland area under irrigation from the Luwombwa River and it's tributaries such as Munte, Musangashi, Mulembo, Sasa and Nyamanda among others. Based on the land uses in the catchment, four classes of water demand were identified as (i) domestic and bulk supply of water (ii) environment or ecological demand (iii) agricultural (irrigation, aquacultural and livestock watering) and (iv) industrial purpose. The catchment population for the Census Years 2000, 2010 and 2022 were 56,013, 67,672 and 90,000 respectively [19] with an average annual population growth rate of 2.45 % and a population density of 24 persons per km^2^. As of 2022, 76.6 % is rural population while 23.4 % is resident in urban areas.

### 2.2 Methods

#### 2.2.1 Rationale for selecting the WEAP model

Several studies have concluded that WEAP is useful for water resources assessment in Southern African catchments providing a holistic view of an entire river basin. For example [20] reported that the Government of Rwanda was developing plans for four selected demonstration catchments using the WEAP modelling tool to introduce integrated land and water management. In Mauritius [21] the WEAP model was used to simulate river systems in 60 catchments to assess the implications of water requirements on national water supply schemes. A number of studies on water availability and management using the WEAP model have been reported with great success in Southern Afr ica [[22], [23], [24]]. In comparison to other hydrological models and for the purpose of this study, WEAP model was adopted due to its proven ability in evaluation and optimisation of complex catchment water resources for supply and demands even under climate change and over long-term and due to its unlimited accessibility in the study region and its data requirement [25,26].

#### 2.2.2 WEAP model setup

The water resources optimisation configuration was performed for demand sites based on their priority level of allocation in WEAP model [27]. In this study, the temporal resolution of the WEAP model input data was configured at monthly time-step for 34 years (1988–2022). The input data comprised of climatic and physical input data collected from different sources (Table S1). The hydrological module implemented during model configuration was the soil moisture method which is based on empirical functions that describe surface runoff, evapotranspiration, interflow, and deep percolation losses for a catchment unit [[28], [29], [30], [31]]. The soil moisture method is based on an algorithm of the one dimensional‐two‐soil‐layer conceptual model (see Fig. S1) and applies a mass balance equation to model catchment hydrological processes [32] as shown in Eq. (1). The advantage of this method is that it allows for the characterization of land use and/or soil type impacts on hydrological processes [33,34] while providing simple realistic ways of water resources modelling of hydrological processes with semi-physical representation [32].

(1) $$Sw_{j}\frac{{dz}_{1,j}}{dt}=P_{e}-PET\left(t\right)k_{c,j}\left(t\right)\left(\frac{{5z}_{1,j}-2z_{1,j}^{2}}{3}\right)-P_{e}\left(t\right)z_{1,j}^{{RRF}_{j}}-f_{j}k_{s,j}z_{1,j}^{2}-\left(1-f_{j}\right)k_{s,j}z_{1,j}^{2}$$

where: ${Sw}_{j}$ is the soil water holding capacity of an area *j* (mm), $z_{1,j}$ represents the relative soil water storage in the soils root zone layer in area *j*, $P_{e}\left(t\right)$ is effective precipitation at time *t* (mm), PET(*t*) is the reference potential evapotranspiration in (mm/day), $k_{c,j}$ is crop coefficient for the area *j*, ${RRF}_{j}$ is the runoff resistance factor for an area *j* while $f_{j}$ represents horizontal and vertical flow partitioning coefficient and $k_{s,j}$ is the saturated hydraulic conductivity of the soil in the root zone layer for an area *j* (mm/time).

**Fig. 2:**
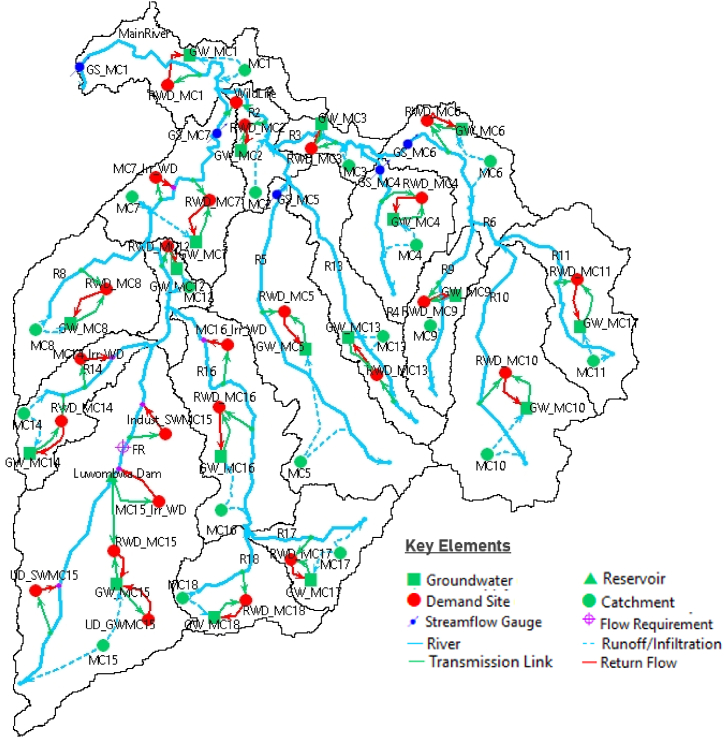
Schematic Configuration of WEAP Model for Luwombwa sub-catchment in the respective 18 micro-catchments, groundwater points.

The sub-catchment was disaggregated into 18 micro-catchments (Fig. 2) where each micro-catchment was treated as a source of water supply with respective demand sites depending on the presence of water use and largely depended on direct river flow abstraction for irrigated agriculture, domestic water use and industrial use. As shown in Fig. 2, the schematic view of the key elements in WEAP Model for the sub-catchment comprised 26 demand sites (red nodes) with respective water allocation priority levels, 18 micro-catchments (green circular nodes), 18 groundwater sources/points (green square nodes) and 5 streamflow gauges (blue nodes). The transmission and runoff/infiltration links were configured accordingly while 1 reservoir (green triangular node) and 1 flow requirement were introduced in micro-catchment number 15 during scenario modelling.

#### 2.2.3 Estimation of catchment water demand

The domestic water demands (DWD) comprised per capita urban and rural water demand estimates based on prorated 17 smallest administrative units: Lulimala, Mpelembe, Chipundu, Luombwa, Chalilo, Chitambo, Muchinka, Mailo, Kabamba, Ibolelo, Muchinda, Nganswa, Lupiya, Musangashi, Lulimala, Chikanda and Munpamazi at ward level to the respective 18 micro-catchments within the sub-catchment. The sub-catchment's average population growth rate estimated from the three recent most census of population for the years 2000, 2010 and 2022 was 2.45 % per annum [19]. The population in the catchment for the year 2022 was 90,313. At an annual growth rate 2.45 % the population is expected to rise to 179,340 by the year 2050 (see Fig. S2). The total annual per capita DWD for each of the 18 micro-catchments is computed using Eq. (2) [[35], [36], [37]].

(2) $$\text{DWD}\;\left(m^{3}\right)=\text{per}\;\text{capita}\;\text{water}\;\text{consumption}\;\left(m^{3}/\text{year}\right)\times \text{micro}‐{\text{catchment}}^{\prime }s\;\text{population}$$

The per capita water consumption rates adopted in this study are 40 and 95 L per capita per day (lcd) for rural and urban population respectively as prescribed by the Zambian Bureau of Standards (ZABS).

The environmental flow requirement in the study area encompassed all forms of catchment ecological water needs under ecohydrological processes, human-nature interactions and factors such as land use-land cover changes on which natural habitats highly depend within the Luwombwa sub-catchment [38]. The sub-catchment water resources allocation for the environmental flow requirement was set to priority two integrating environmental factors such as land use-land cover and climate change (Table S2).

Agricultural (rain-fed, irrigated, aquacultre and livestock) water demand in the catchment was estimated based on field data collected from the water users coupled with remote sensing data [[39], [40], [41]]. The irrigation water demand (IWD) in Million m^3^ (Mm^3^) which includes both supplementary and complementary irrigation for rainfed and winter crops respectively was estimation using Eq. (3) [[42], [43], [44]].

(3) $$\text{IWD}\;\left(Mm^{3}\right)=\left[{\text{ET}}_{0\;}\left(\frac{\text{mm}}{\text{day}}\right)\times \text{Average}{\;K}_{c}\right]-\left[\text{Rainfall}\;\left(\text{mm}\right)\times \text{Effective}\;\text{Rainfall}\;\left(\%\right)\right]\times \left[1+\left(1\;–\;\text{Irrigation}\;\text{Method}\;\text{Efficiency}\;\left(\%\right)\right)\right]\times \left[\text{Crop}\;\text{Area}\;\left(\text{ha}\right)/100,000\right]$$

where: ${\text{ET}}_{0\;}$ is Reference Evapotranspiration rate (mm/day), and K_c_ is the Crop Coefficient Factor

Industrial water demand (InWD) for industrial use was obtained from the national water resources regulator - Water Resources Management Authority (WARMA) and incorporated into the study. The main purpose of this type of water use was mineral processing with an estimated demand of 1.2 Million m^3^ per year. For water allocation modelling InWD was ranked under priority level 4 in the sub-catchment (Table S2).

#### 2.2.4 WEAP model performance evaluation

The model was calibrated and validated using the observed and simulated streamflow closely evaluated against acceptable thresholds of three statistical objective functions (Eqs. (4), (5), (6))) as suggested by Moriasi et al. [45]. The Nash–Sutcliffe model efficiency coefficient (NSE) greater than 0.50 (50 %) was considered satisfactory for assessing how the model fitted simulated streamflow compared to the observed streamflow data in the sub-catchment level [46,47], Coefficient of Determination (R^2^) being the correlation between the observed and model simulated streamflow with values approaching 1 (100 %) indicate high model performance and the Percent Bias (PBIAS) indicated whether the model is under or overestimating simulated flows and ranges between −25 % and +25 % where a positive (+) indicates flow underestimation and negative (−) value indicates model streamflow overestimation [45,48,49]. Model calibration and validation highly relied on observed streamflow data from five hydrometric stations with observation record from 1989 to 2022 within the sub-catchment.

(4) $$\text{NSE}=1-\frac{\sum_{t=1}^{T}{\left({Q_{s}}^{t}-{Q_{0}}^{t}\right)}^{2}}{\sum_{t=1}^{T}{\left({Q_{0}}^{t}-{\bar{Q}}_{0}\right)}^{2}}$$

(5) $$\begin{array}{cc} R^{2}= & \frac{\sum_{t=1}^{T}{\left[\left(Q_{o}^{t}-{\bar{Q}}_{o}^{t}\right)\left(Q_{s}^{t}-{\bar{Q}}_{s}^{t}\right)\right]}^{2}}{\sum_{t=1}^{T}{\left(Q_{o}^{t}-{\bar{Q}}_{o}\right)}^{2}\sum_{t=1}^{T}{\left(Q_{s}^{t}-{\bar{Q}}_{s}\right)}^{2}} \end{array}$$

(6) $$PBIAS=\frac{\sum_{t=1}^{T}\left(Q_{s}^{t}-Q_{o}^{t}\right)\left(100\right)}{\sum_{t=1}^{T}\left(Q_{o}^{t}\right)}$$

In Eqs. (4), (5), (6)), ${\bar{Q}}_{0}$ is the mean of the observed streamflow, while ${Q_{s}}^{t}$ and ${Q_{0}}^{t}$ is simulated and observed streamflow at time t, respectively.

With satisfactory model performance achieved, scenario analysis on future water allocation uncertainty in addressing “what if” questions was implemented. What if commercial agricultural irrigation expands in the catchment, what if climate change pose temperature rise and reduced rainfall amounts in the catchment were some of the specific questions of uncertainty addressed during scenario modelling in the study area [[50], [51], [52]].

#### 2.2.5 Future climate change data downscaling

Future scenario to facilitate climatic change modelling was obtained from four statistically downscaled and bias-corrected GCMs data comprising temperature and precipitation through CMIP6 covering the ensembles of SSP126, SSP245 SSP370 and SSP585 [13,51,53,54]. The GCM climate projection models are regridded on a common of 1:1 grid using a bilinear interpolation before the multimodel analysis with their climatologies are calculated with the first member-usually r1i1p1f1. In this study we adopt similar GCM selection methodologies reported by McSweeney et al. [17] along with improved rank score-based and criteria method using the historical sub-catchment's collected climatic data from 1987 to 2022. This process facilitated the development of focused suitable, significant and representativeness of high-resolution climatic data from the GCMs in the region and sub-catchment while excluding identified poor representation of model data from ensemble members such as MOHC-HadGEM3-GC31-LL and NIMS-KMA-KACE-1-0-G. The excluded ensemble members showed a tendency of overestimation in average temperature which was part of the limitation in direct application of GCM climatic data to the micro-catchments apart from their coarseness in resolution-usually continental scale making them responsible for large uncertainty. Based on the improved rank score-based with criteria method performed on 15 GCM members, four ensemble members; MIROC6, CNRM-CM6-1, MRI-ESM2-0 and MPI-ESM1-2-LR were selected as they showed better climatic data simulation performance and seasonality representation in the sub-catchment compared to other ensembles from GCMs assessed. The study addressed bias correction through the delta change approach [55] with emphasis on statistical downscaling of catchment climate data from GCMs. The catchment's analysed future projections for temperature and rainfall variability in this research was based on SSP370 scenario. The GCM downscaling climate data preprocessing was conducted at micro-catchment level where estimates of rainfall and temperature were calculated using the areal average technique in the ArcGIS environment version 10.8 alongside other tools such as Microsoft Excel for each of the 18 micro-catchments for future projection from the baseline. The GCM dataset was obtained from NASA Earth Exchange (NEX) Global Daily Downscaled Projections (NEX-GDDP). The climate dataset was developed from GCMs runs performed by CMIP6. Table 1 shows the GCM used in this work and their respective resolutions.

**Table 1:** The four GCMs from CMIP6 applied in climate change Scenario analysis.

Research Center	Country	GCM	Grid resolution (lat., long.)
Japan Agency for Marine-Earth Science and Technology (JAMSTEC), Atmosphere and Ocean Research Institute, The University of Tokyo (AORI), National Institute for Environmental Studies (NIES), and RIKEN Center for Computational Science (R-CCS, MIROC)	Japan	MIROC6	1.39° × 1.406°
Centre National de Recherches Meteorologiques (CNRM), Centre Europeen de Recherche et de Formation Avancee en Calcul Scientifique (CERFACS)	France	CNRM-CM6-1	1.40° × 1.406°
Meteorological Research Institute (MRI)	Japan	MRI-ESM2-0	1.113° × 1.125°
Max Planck Institute for Meteorology (MPI-M)	Germany	MPI-ESM1-2-LR	1.39° × 1.41°

#### 2.2.6 Catchment water allocation under different climate scenarios

The research adopted the WEAP model for catchment water resources demand and allocation analysis. According to the model, water resources demand site was based on priorty of allocation of supply relative to all other demands within the model. In this case, the demand site priorities ranged from one (1) to ninety-nine (99), where one refers to the highest priority and 99 the lowest [56]. The assessment of catchment water resources allocation through the estimation of available water resources in reasonable and practical framework within the sub-catchment was conducted through water use demand analysis by setting water use priorities based on the Zambian Water Resource Management Act No. 21 of 2011. According to WARMA [57], the major purposes of water use are: (i) domestic and non-commercial; (ii) environmental; (iii) training and research; (iv) municipal; (v) agricultural (commercial irrigation, aquaculture and livestock); (vi) industrial; (vii) hydropower generation; (viii) mining; (ix) navigational; (x) bulk water supply; (xi) others such as abstractive recreational water uses. However, for Luwombwa sub-catchment the existing water uses are domestic and bulk water supply, environmental, agricultural and industrial with water allocation priorities of 1, 2, 3 and 4 respectively.

**Fig. 3:**
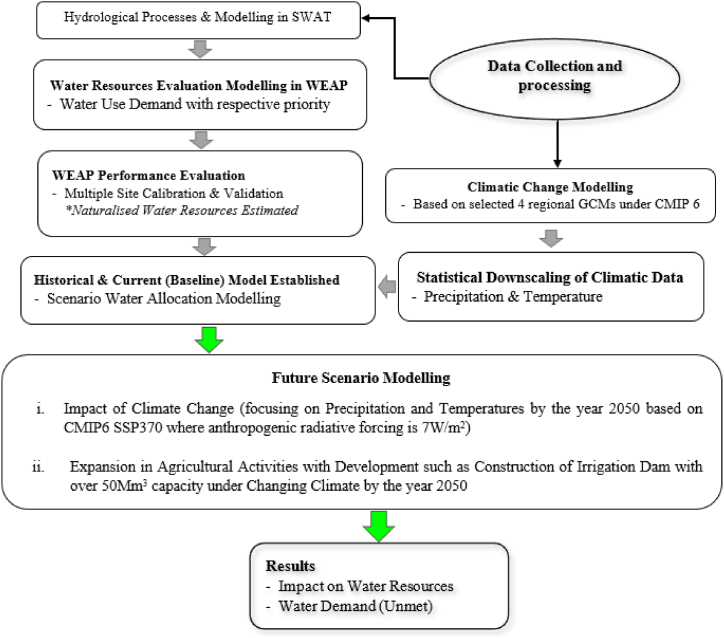
Methodological Framework of the Study data acquisition and hydrological modelling and analysis of water demand and allocation.

Based on the framework (Fig. 3), the analysis approach involved several processes including estimation of catchment water availability through hydrological modelling dissagregated at micro-catchment level using the calibrated and validated Soil Water Assessment tool (SWAT) model [47, under review]. The evaluation of optimal water resources allocation in historical and current year period for future projection scenario in the calibrated and validated WEAP model. Multiple site calibration and validation of streamflow in WEAP using five hydrometric stations in respective dissagregated micro-catchments equivalent to Hydrological Response Units (HRUs) were achieved. Future catchment water resources available for efficient allocation were simulated based on four statistically downscaled and bias-corrected Global Climate Models (GCMs) climatic data sourced from Coupled Model Intercomparison Project Phase (CMIP6). This was done for the climatic change senarios anaysis such as assessment of the impact of increased temperatures with reduction in catchment rainfall amount along side anthropogenic changes including land use change, agricultural expansion and population increase on catchment water resources. Evaluation of historical, current and future catchment water resources were then analysed based on water balance approach and unmet demand. The general water balance was applied based on Eq. (7) [16].

(7) P+ExtIn=ET+Outflow+ABST±ΔS

*P* is the catchment precipitation in Mm^3^, *ExtIn* is external inflows from other sources such as flows from interbasin transfer in Mm^3^, ET is the catchment evapotranspiration Mm^3^, *Outflow* is streamflow out of the catchment Mm^3^, *ABST* represents all water resources abstractions form the catchment in Mm^3^ and ***ΔS*** is the change in catchment soil water storage in Mm^3^.

## 3 Results and discussion

### 3.1 Evaluation of model performance

#### 3.1.1 Sensitivity analysis

The acceptable and efficient model for the study area was achieved in three sequential steps which included senstitivity analysis, parameter calibration and model validation. Table 2 presents results of the seven most appropriate and sensitive parameters for the sub-catchment. The order of model parameter sensitivity is indicated from most to least sensitive and were found to be attributed to the sub-catchment's hydrological processes mostly influencing the generation of streamflow [28,29]. In the study area, the runoff resistance factor (rrf) responsible for controlling surface runoff and related to factors such as leaf-areal-index and land slope was the most sensitive parameter from its default value of two (2) to the model fitted value range between 1.5 and 7 in the sub-catchment. The soil water capacity (swc) and crop coefficient (kc) ranked second and third in sensitivity respectively while the preferred flow direction (pfd) was the least sensitive parameter (Table 2). Fernández-Alberti et al. [34] in their study indicated similar sensitive parameters including deep-water capacity, runoff resistance factor, deep conductivity and preference flow direction when they applied a landuse module in WEAP to evaluate the water resources of a natural protected area as a provider of ecosystem services in southern Chile. Similary, Tena et al. [49] calibrated a WEAP model in Semi-Arid Bweengwa, Kasaka and Magoye sub-catchments of southern Zambia indicating similar sensitive parameters. The fitted range of runoff resistance factor provide an indication of normally distributed slope in the sub-catchment.

**Table 2:** Model parameter estimation and sensitivity analysis.

Sensitivity rank	Parameter Name	Description	Initial Parameter range	Default parameter value	Model Fitted optimal parameter Range
1.	Runoff Resistance Factor (rrf)	Controls surface runoff. Related to factors such as leaf-areal-index and land slope (m/m).	0–1000	2	1.5–7
2.	Soil Water Capacity (swc)	Effective soil water holding capacity of upper soil layer (mm).	>0	1000	900–1500
3.	Crop Coefficient (kc)	Crop coefficient relative to the reference crop evapotranspiration.	>0	1	0.4–1.05
4.	Deep Water Capacity (dwc)	The effective water holding capacity of lower, deep soil layer (mm).	>0	1000	800–1100
5.	Root Zone Conductivity (rzc)	Root Zone hydraulic conductivity which will be partitioned according to preferred flow direction between interflow and flow to the lower soil layer (mm/month).	Varies among land class type	20	15–50
6.	Deep layer Conductivity (kd)	Conductivity rate of the deep layer at full saturation which controls baseflow transmission (mm/month).	>0.1	20	20
7.	Preferred Flow Direction (pfd)	Responsible for effective water hold capacity of upper soil layers in the catchment (mm)	0–1000	0.15	0.17–0.8

#### 3.1.2 Calibration and validation

**Fig. 4:**
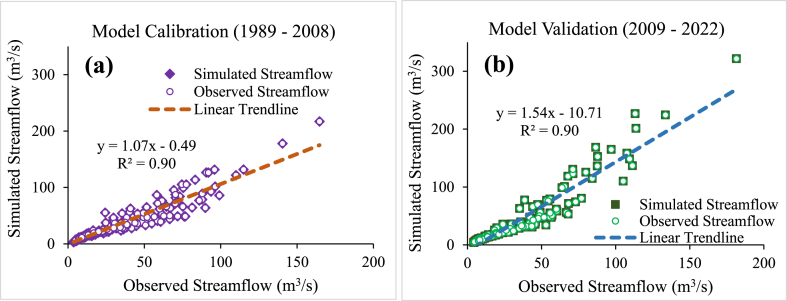
Observed and simulated average monthly streamflow for the calibration period (1989–2008) (a) and for the validation period (2009–2022) (b).

The WEAP model was calibrated and validated on a monthly time-scale with observed streamflow at five respective hydrometric stations within the sub-catchment from (1989–2008) and (2009–2022) respectively. The results evaluated through statistical objective functions are presented in Table 3 with acceptable thresholds attained during model calibration and validation thereby providing an indication of good agreement between observed and simulated streamflow. In addition, the results in Fig. 4(a) and (b) shows the scatter plots of observed and simulated streamflow for the calibration and validation periods with acceptable statistical values for the model in the entire sub-catchment. In this study we performed multiple site calibration and validation and achieved satisfactory objective functions R^2^ at 92 %, NSE at 82 % and PBIAS at 6.9 % for the entire sub-catchment's model performance. This confirms the indication of WEAP's capability in reproducing catchment hydrological processes by Tena et al. [16] who reported R^2^ of 97 % and NSE of 64 % to achieve satisfactory model fit in the Chongwe River Catchment. Moriasi et al. [45] in their study suggested that better model performances are realised if the values of R^2^ is close to unity (one), NSE has values greater than 0.5 and closer to one and PBIAS has small values ≤ ±25. A hydrological model is considered calibrated for streamflow if the statistics for monthly time step NSE >0.50, PBIAS ≤ ±15 % and R^2^ > 0.50 indicating a satisfactory model [45,58,59]. In similar studies Asghar et al. [60] reported NSE and R^2^ values of 0.85 (85 %), 0.86 (86 %), 0.89 (89 %, and 0.87(87 %) for their model based on monthly calibration and validation periods between the measured and simulated streamflow in the central Indus basin.

**Table 3:** Evaluation of objective functions calibration and validation statistics - NSE, R^2^ and PBIAS – for the WEAP Model at the five hydrometric stations (6–512, 6–513, 6–514, 6–516, and 6–525).

Station ID	River	River gauging station	Area coverage (km^2^)	Calibration period *(1989 to 2008)*	Validation period *(2009 to 2022)*
6-625 *(main outlet)*	Luwombwa	Kabesa Village	7363	NSE: 0.85	NSE: 0.85
				R^2^: 0.90	R^2^: 0.90
				PBIAS: 14 %	PBIAS: −2.9 %
6–513	Mulaushi	Wasa Road Bridge	327	NSE: 0.82	NSE: 0.81
				R^2^: 0.87	R^2^: 0.81
				PBIAS: 10 %	PBIAS: 3 %
6–514	Kasanka	Old Pontoon	1191	NSE: 0.79	NSE: 0.74
				R^2^: 0.88	R^2^: 0.83
				PBIAS: 11 %	PBIAS: −12 %
6–516	Luwombwa	Luwombwa Lodge	3712	NSE: 0.88	NSE: 0.72
				R^2^ : 0.89	R^2^ : 0.90
				PBIAS: −1.7 %	PBIAS: −14 %
6–512	Mulembo	Tuta Road Bridge	1646	NSE: 0.86	NSE: 0.89
				R^2^: 0.9	R^2^: 0.91
				PBIAS: 0.32	PBIAS: −11 %

Finally, Gedefaw et al. [1] conducted an assessment of water resources allocation systems under irrigation expansion and climate change scenario in the Awash River Basin of Ethiopia and achieved average values of R^2^ at 82 % and NSE at 74 % indicating a good WEAP model performance. Therefore, the achieved model performance for the Luwombwa sub-catchment indicate model reliability and forms the basis for future scenario projection and analysis.

**Fig. 5:**
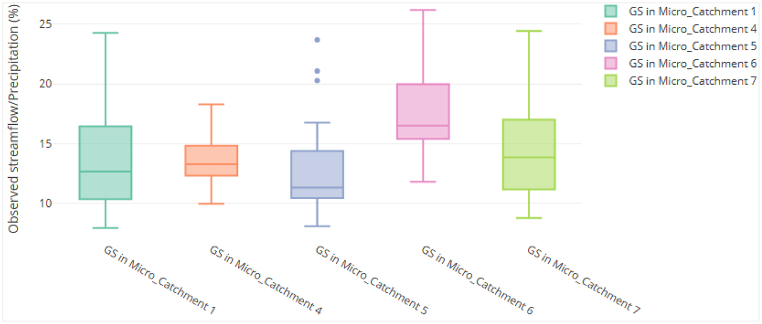
Pecentage runoff (%) estimated from respective Hydrometric Stations (GS) in Luwombwa sub-catchment.

The average percentage runoff of 15.5 % was estimated in the entire sub-catchment where as the range 11 %–20 % was derived using the five respective hydrometric stations (Fig. 5). These results are comparable to the percentage runoff range 0.011 (1.1 %) – 0.35 (35 %) obtained by Machado et al. [61] when determining runoff coefficient in five experimental catchments based on analysis of precipitation and flow events in São Paulo. It was revealed that the higher the value, the more the runoff which is an indication on catchment land use land cover and vegetation diversity. Therefore, the estimated result from calibration and validation for percentage runoff at 15.5 % is acceptable in the Luwombwa sub-catchment as forest cover at 44 % is the dominant landuse while rapaid development including urbanisation, cropland, graze land share the rest of the land use-land cover characteristics.

The sub-catchment's water balance based on Eq. (7) in volumetric terms per year for the calibration period was estimated at 592.37 Mm^3^ and 407.19 Mm^3^ during the validation period. The water balance for the entire Luwombwa sub-catchment was 875.58 Mm^3^ with a positive storage indicating water availability in the sub-catchment (Table 4). According to Table 4 and results based on Eq. (7) from the entire clibaration and validation periods the largest sub-catchment water loss was through evaportranspiration at 63 % of the precipitation on average indicated by Mwelwa et al. [47, under review]. The catchment percentage water losses through evapotranspiration are similar in the region and range 64 %–69 % indicated in the study by Desta and Lemma [62]. The annual water abstraction for various water uses was estimated at 11 % in the entire Luwombwa sub-catchment.

**Table 4:** Average Catchment Water Balance for the Calibration (1989–2008) and Validation (2009–2022) periods.

**Water Balance Components**	**Calibration Period**	**Validation Period**	Average Catchment Water Balance
Precipitation (Mm^3^)	8018.54	8216.14	8106.9
Streamflow (Mm^3^)	1192.63	1295.11	1229.38
Evapotranspiration (Mm^3^)	5932.06	5911.48	5098.1
Abstraction (Mm^3^)	301.48	602.36	903.84
**Storage (Mm**^**3**^**)**	**592.37**	**407.19**	**875.58**

### 3.2 Climate change analysis and water allocation

#### 3.2.1 Climate change analysis

**Fig. 6:**
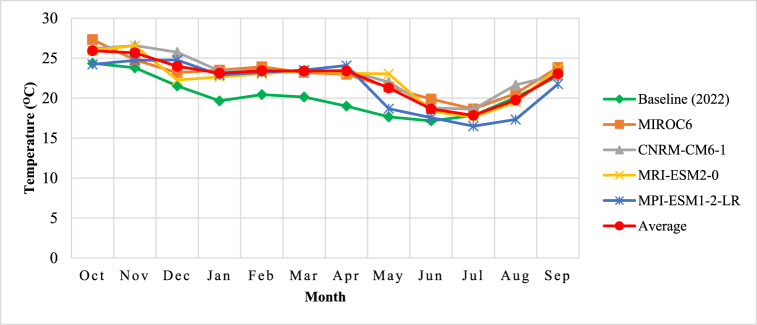
Comparison of projected monthly temperatures derived from different GCM with baseline temperature.

In this study, the important climatic variables – rainfall and temperature statistically downscaled from four selected GCMs under ensembles in SSP370 within CMIP6 revealed a considerable reduction in average annual rainfall amounts by 4.5 % and increase in average maximum temperatures by 9.3 % (4 °C) in the sub-catchment by the year 2050. The upward shift in temperature is depicted in Fig. 6 presented interms of hydrological year from October (Oct.) to September (Sep.) the following year where the average devried from GCMs is high [[63], [64], [65]]. The average maximum and minimum temperatures by 2050 varied between 28 °C and 17.8 °C while the baseline indicated the variation of 24 °C and 17.1 °C respectively. In climatic change related studies, Almazroui et al. [66] analysed a multi-model ensemble based on 27 CMIP6 model and their results indicated 4.4 °C rise in temperature over Africa and 4.1 °C mean temperature rise over South Eastern Africa (SEAF). Similar results were also indicated by Moses [67] and Nkemelang et al. [6] in southern Africa. This rise in temperature may impact negatively especially on water resources availability where nature and livelihood depend. In addition, this effect promotes water loss through increased evapotranspiration and heat stress thereby affecting available water in the sub-catchment [68].

Climate change analysis on the other hand showed a decreased in sub-catchment rainfall amount with the mean annual rainfall of 1054 mm at baseline to 1007 mm the corresponding anticipated average by the year 2050 as projected under selected GCMs. In other studies, Fant et al. [69] reported similar results in the region with ±10 % decrease in precipitation even with the application of large (6800) essembles of climate change related to Greenhouse gas emission policies. This clearly represents the distribution of impacts on water resources and regional climate uncertainty among others within the Zambezi river catchment encompassing four major countries including Malawi, Mozambique, Zambia and Zimbabwe. The resulting decrease in average rainfall and increase in average temperature shows the likelihood of sub-catchment's vulnerability to drought conditions hence the need to preserve, secure and manage sub-catchment water resources for future sustainability for both local rivers and regional river systems [14,[70], [71], [72]].

#### 3.2.2 Water allocation under scenarios

**Fig. 7:**
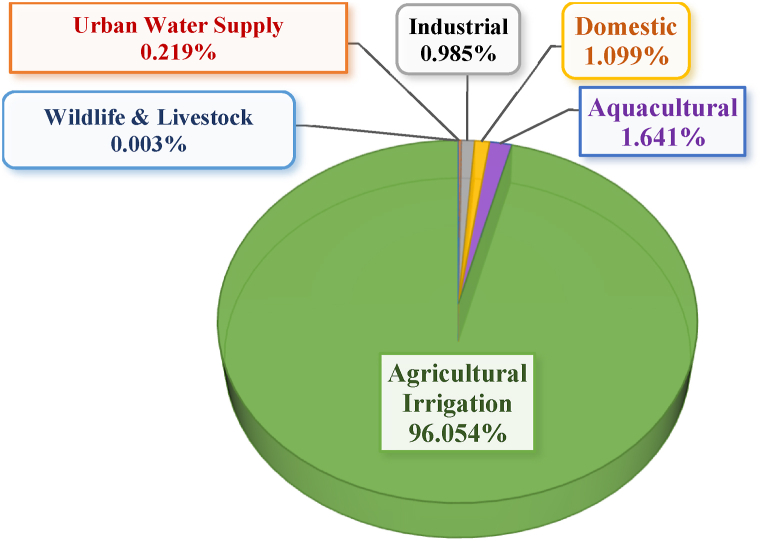
Estimated baseline year-2022 Water Demand by Sector in Percentage within the Luwombwa sub-catchment.

The six sectoral water demand modelled in the sub-catchment results at baseline revealed that irrigated agericulture had 106.8 Mm^3^ water consumption representing 96.1 % of the total 111.2 Mm^3^ sub-catchment's water use demand and was the highest water user. Aquaculture water demand estimated at 1.8 Mm^3^ was the second large water use representing 1.6 % while the rest of the demands were below 1.5 % as shown in Fig. 7. The percentage representation margin or gap of type of water use being wide indicating that the agricultural sector is the dominant sub-catchment's water resources user within the Luwombwa sub-catchment a similar results were also reported by Portmann et al. [73]. Khan et al. [74] also established agricultural irrigation through global monthly sectoral water use to have larger withidraws and consumption especially under SSP-RCP GCM combination by sector as a dominant water user.

**Fig. 8:**
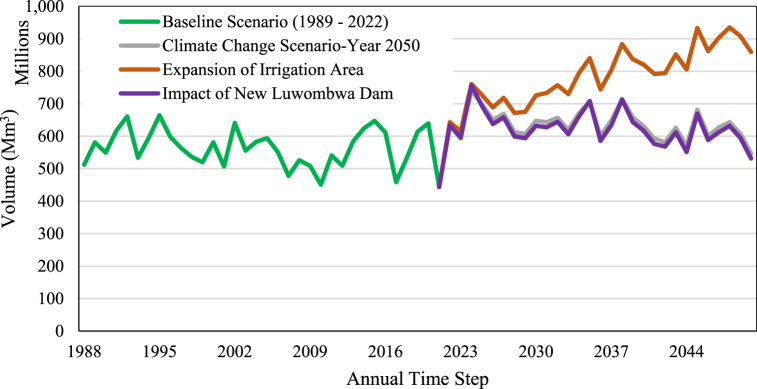
Luwombwa sub-catchment water resources variability under selected scenarios.

In terms of water allocation, the sub-catchment water resources were assessed under different two main scenarios, the baseline (2022) and climate change (2050) scenario. The results for water resources unment demand estimated at 562 Mm^3^ and 683 Mm^3^ per year for the baseline and climate change scenario respectively. The climate change scenario applied to the 18 micro-catchments comprised of components which included, water demand with a predicted population growth rate at 2.45 % per annum resulting in unmet demand of 626 Mm^3^. The impact of the irrigation dam with estimating 627 Mm^3^ together with environmental flow requirements as ecological streamflow to downstream and expansion of irrigation area under climate change scenario resulted in 787 Mm^3^ unmet demand, causing a significant impact of the sub-catchment's water resources in the face of future climate change. The sub-catchments high unmet demands were for agricultural irrigation water requirements. The effects of the aforementioned unmet demand results for the whole sub-catchment translate to 20 % decrease in the sub-catchment's water resources in the near future by 2050 and also to the increased demand from the sectors (Fig. 8). Hassan et al. [75] and Höllermann et al. [76] assessed future water demand and supply balancing due to an anticipated decrease in water resources by 2040.

The near future with forcing SSP370 [77,78] persistence show that the unmet demand under scenario of catchment agricultural expansion by 2 % (Fig. 8.) would be significant thereby causing lots of pressure on the Luwombwa river system. However, if this scenario is combined with mitigation measures such as construction of dam to store water during the rainy season for later use;- the impact is not significant compared to the scenario with direct abstraction from the river system. The climate change projections indictaed temperature increase and decrease in catchment rainfall amount under the SSP370 conditions coupled with population increase has less bearing on the catchment water resources which is also true when combined with scenario on dam construction along with flow requirement implementation in the catchment. Therefore, the combined scenarios of climate change with anthropogenic activities such as deforestation and industrialization for the near future show negligible impact on water resources except for the expansion in the agricultural scenario. This means that with the prevailing climatic change in the near and far future, expansion of irrigated agriculture has to be managed and mitigated in the sub-catchment to ensure sustainability of catchment water resources availability and this requires an integrated approach [79] due to climate change uncertainties in the region. Under the climate change and socioeconomic development impact, the study indicates that Luwombwa sub-catchmnet will experience a general downward trend of annual streamflow, a likely increase in the value of water due to anticipated increase in agriculture, aquaculture, and domestic water demand and water scarcity in the future.

#### 3.2.3 Adaptive water resources management and policy making under changing climate

Under changing climate, the study highlights the critical importance of water resources management strategies that should consider such options as improved resilience of water systems through water conservation, water reuse and water saving technologies; identifying water-related risks and reducing vulnerabilities by investing in water education and awareness programmes; development of water management plans that take into account climate change and the needs of different stakeholder groups. Enhancement of regulation and compliance monitoring of water use among the competing water users by installation of real-time metering facilities such as Advanced Metering Infrastructure (AMI) technologies at respective main water abstraction points and expansion of telemetric hydrometric network on the sub-catchment's river system to promote and ensure future water resources accountability and sustainability in the management and allocation of water resource. Our study underscores the need for policy-makers to develop and implement comprehensive policies that address the impacts of climate change as well as taking into account the needs of different users. Future researchers should assess irrigation methods practiced in the sub-catchment as noticed from Fig. 7 providing an indication of the need to emphasise and promote smart irrigation practices for example through smart irrigation sensors in optimising outdoor water use especially in the cases of drought would improve the soil texture, increase the soil water retention capacity. This will lead to better soil and water conservation, resilience and increased crop yield as a specific amount of water is applied to the crops-based water deficit detected in the soils and rainwater harvesting suitability analysis with technologies. Study conducted with the application of soil moisture/irrigation sensors in the semi-arid northern Ethiopia indicated that crop yield significantly increased around soil and water conservation structures due to runoff retention and enhanced soil moisture [80]. Rainwater harvesting is key in promoting adaptive measures for the future climate change, just like the planned Luwombwa dam- and construction of more dams, ponds and diversion weirs needed to mitigate water scarcity in the future are recommended especially in the middle parts of the micro-catchments 10, 11, 17 and 18. Climate change is expected to induce extreme rainfall and flooding [81]. Therefore, water harvesting, sustainable utilization and management of water resources is crucial in the face of climate change. Restoration of soil organic matter, mainstreaming adaptation in climate change at national level plays a vital role through, increasing awareness which is a crucial adaptive measure to ensure equitable water allocation under changing climate in the sub-catchment [72].

## 4 Conclusions

The study evaluated catchment water resources allocation under climate change and anthropogenic development by utilising the WEAP model in the semi-arid Luwombwa sub-catchment, Zambia. A three phased approach of model development was conducted in historical, current status (baseline) and future state informing scenario modelling. Through historical climatic and water demand data, the model was calibrated and validated with satisfactory ranges of objective functions (R^2^, NSE and PBIAS) together with catchment parameter sensitivity analysis to enabled estimation of catchment water balance and availability under various existing competing water users. The determination of the water balance formed as baseline for future scenario analysis. The factors that cause pressure on catchment water resources such as population growth, agricultural expansion, industrialization and climatic variability were incorporated in the water allocation model to assess their impact on future water resources avaialability and allocation. Results for both the near future (2050) scenarios showed variations in future water availability with significant decrease in catchment water resources by 20 % leading to unmet demands while mitigation measures if adapted shows sustainability of the available water resources in the catchment.

The research simulated uncertainty due to effects of climate change on catchment water resources through statistically downscaled climate data from CMIP6 for the near future (year 2022–2050) coupled in WEAP focusing on regional rising catchment temperatures estimated by 9.3 % and a declining rainfall amount by 4.5 % with persistence of El-Nino conditions. The magnitudes of change of SSP370 were not significant to cause a change on water availability in the future unless couple with land use land cover change such as expansion of irrigated agriculture to mitigate the impact of climate change. This gives direction for decision and policy-makers to maintain and manage the existing water resources for future to reduce threats due to climate change effects and land use change that led to the expansion of irrigation. In addition, the negative impact of climate change and uncertainty on sub-catchment's water resources availability revealed in the study, offer insight to policy-makers including stakeholders and line ministries within governments on drafting preparedness early action plans in various sub-catchments to ensure equitable future water resources allocation and development of sustainable water resources management plans. The study entails the need for developing and implementating water-saving technology such as improved irrigation system in the sub-catchment, water allocation policy, and restoring water resources for sustainable utilization in the face of climate change.

## CRediT authorship contribution statement

**Dickson Mwelwa:** Writing – original draft, Methodology, Investigation, Formal analysis, Data curation, Conceptualization. **Phenny Mwaanga:** Writing – review & editing, Visualization, Supervision. **Alick Nguvulu:** Writing – review & editing, Visualization, Supervision. **Tewodros M. Tena:** Writing – review & editing, Visualization, Supervision. **Gebeyehu Taye:** Writing – review & editing, Visualization, Supervision, Conceptualization.

## Data availability statement

Data will be made available on request.

## Declaration of competing interest

The authors declare that they have no known competing financial interests or personal relationships that could have appeared to influence the work reported in this paper.
