# Supplementary material for: Assessment of catchment water resources allocation under climate change in Luwombwa sub-catchment, Zambia
Source: Heliyon. 2024 Oct 30;10(21):e39962. doi: 10.1016/j.heliyon.2024.e39962 (PMC11567020; doi:10.1016/j.heliyon.2024.e39962)
Supplement: Multimedia component 1 [file mmc1.docx]

**Supplementary Materials**

Table S1: Data sources for historical and baseline model configuration

| Data | Data Source |
| --- | --- |
| Precipitation, temperature (min/max), relative humidity, wind speed and solar radiation (1980 -2024) | Zambia Meteorological Department (ZMD),  CHIRPS - <https://climateserv.servirglobal.net/map> , |
| Local Soil Map | Zambia Agriculture Research Institute - Mount Makulu Research Station. |
| Land cover Land use | National Remote Sensing Center (NRSC) of Zambia and |
| Observed Streamflow Data | Water Resources Management Authority (WARMA), Zambia |

**Table S2:** Allocation Priorities of Luwombwa Catchment Water Use

| Water Use or Purpose | Priority level |
| --- | --- |
| Domestic and Bulk water supply | 1 |
| Environment | 2 |
| Agricultural | 3 |
| Industrial/Mining | 4 |


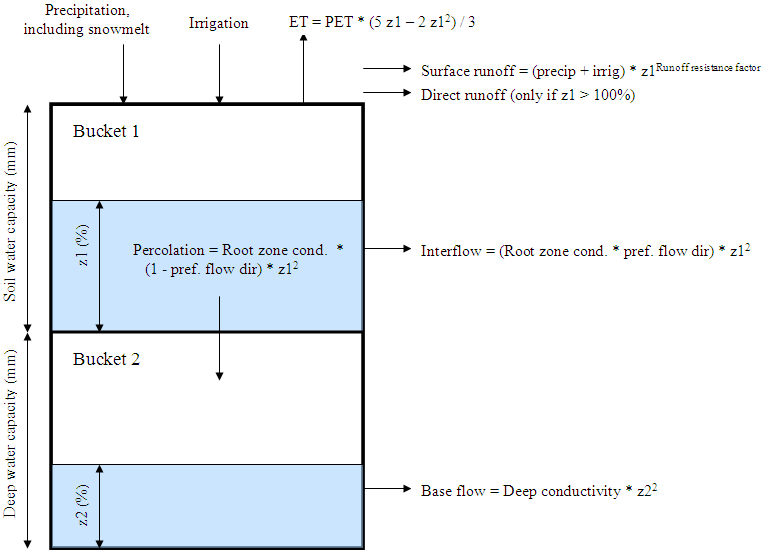


Fig. S1. Conceptual Model representation of Soil Moisture method in WEAP (Source: Sieber and Purkey, 2015)

Fig. S2. Observed and Projected Population in the Catchment
